# Skin thickness score as a surrogate marker of organ involvements in systemic sclerosis: a retrospective observational study

**DOI:** 10.1186/s13075-019-1919-6

**Published:** 2019-05-28

**Authors:** Kazuki M. Matsuda, Ayumi Yoshizaki, Ai Kuzumi, Takemichi Fukasawa, Satoshi Ebata, Shunsuke Miura, Tetsuo Toyama, Asako Yoshizaki, Hayakazu Sumida, Yoshihide Asano, Koji Oba, Shinichi Sato

**Affiliations:** 1 Department of Dermatology, The University of Tokyo Graduate School of Medicine, 7-3-1, Hongo, Bunkyo-ku, Tokyo, 1138655 Japan; 2 Department of Biostatistics, School of Public Health, The University of Tokyo Graduate School of Medicine, Tokyo, Japan

**Keywords:** Systemic sclerosis, Modified Rodnan total skin thickness score, Interstitial lung disease, Regression analysis

## Abstract

**Background:**

Previous studies have shown the relationship between higher skin thickness score and the existence of organ involvements in systemic sclerosis (SSc). Here, we firstly investigated the correlation between skin thickness score and quantitative measurements of each organ involvement in Japanese patients with SSc.

**Methods:**

All Japanese SSc patients hospitalized to our clinic for initial evaluation of SSc were selected. Skin thickness was evaluated by modified Rodnan total skin thickness score (mRSS). Relationship between mRSS and prevalence or incidence of organ involvements was examined by logistic analyses. Correlation between mRSS and quantitative measurements of organ involvements was examined by correlation analyses and regression analyses.

**Results:**

We recruited 198 patients into our study. The mean disease duration was 7.3 years with the mean follow-up duration of 3.2 years. Multivariate logistic regression analyses revealed that higher mRSS is related to higher prevalence of interstitial lung disease (*P* < 0.05), restrictive impairment (*P* < 0.01), and diffusion impairment (*P* < 0.05) of the lung. Correlation analyses revealed mRSS negatively correlates with forced vital capacity (*P* < 0.001) and diffusing capacity (*P* < 0.001) of the lung. Correlation between longitudinal change of mRSS and that of forced vital capacity (*P* < 0.05) or diffusing capacity (*P* < 0.001) of the lung was also demonstrated.

**Conclusions:**

Skin thickness score significantly correlates with quantitative measurements of lung involvement in Japanese patients with SSc.

## Background

Systemic sclerosis (SSc) is a connective tissue disease characterized by fibrosis, vascular injury, and immunological abnormality across multiple organs [1]. Variable symptoms of SSc are the reflection of each organ involvement by different aspects of the pathology, which makes it difficult to grasp the whole picture of the disease. For example, fibrosis of the dermis causes skin sclerosis [2], while alveolar fibrosis causes systemic sclerosis-related interstitial lung disease (SSc-ILD) [3]. In addition, vascular injury in the extremities causes digital ulcer [4], renal involvement triggers SSc renal crisis (SRC) [5], and impairment of the pulmonary arteries causes pulmonary hypertension [6]. The reflection of immunological abnormality is a variety of autoantibodies detected from the sera of SSc patients [7, 8].

Effective treatment differs by each organ involvement, which requires clinicians to combine multiple therapeutic modalities to treat SSc patients. Some medications have pivotal effect on each organ involvement, making the treatment further difficult. For instance, systemic corticosteroids are used in treating skin fibrosis and SSc-ILD [9, 10], while high-dose corticosteroids are known as a risk factor of SRC development [11, 12]. Calcium channel blockers are sometimes used for treating Raynaud’s phenomenon in SSc patients [13], but they worsen the symptoms of gastroesophageal reflux disease (GERD) [14]. To optimize the combination of such multiple therapeutic modalities, evaluation of severity and disease activity for SSc patients should be multidimensional. Skin thickness is one of such measurements that have long time been used and evaluated as a barometer that mainly reflects the aspect of fibrosis. It has been revealed that tighter skin is a predictive factor of death [15], heart involvement [16], muscle involvement [17], and development of SRC [18, 19]. In addition, higher skin thickness score is related to severer function disability of SSc patients measured by health assessment questionnaire disability index [20, 21]. It has also been reported that rapid increase in skin thickness score is a predictor of higher incidence of early death and SRC [22].

Modified Rodnan total skin thickness score (mRSS) is one of the established methods to examine the skin thickness of SSc patients. It is a semiquantitative, noninvasive, and rapid method to measure the skin thickness with high reproducibility [18], which makes it widely used both in clinical trials and real clinical practice [23]. Previous studies have investigated the relationship between incidence of disease-related events or presence of organ involvement and skin thickness that was quantitatively evaluated [15–19]. However, almost all of them targeted Caucasians, Hispanics, or African American patients.

Herein, we investigated the correlation between mRSS and quantitative measurements of organ involvement by retrospective observation of Japanese SSc cohort. Our goal is to evaluate the utility of mRSS as a quantitative barometer of other organ involvement in SSc patients.

## Methods

### Subject patients

We included all the patients with SSc hospitalized to our clinic since May 2011 until April 2018. All the new patients arrived at our clinic receives initial evaluation for SSc, including physical examinations, laboratory tests, high-resolution computed tomography (HRCT) scanning of the lung, pulmonary function tests, transthoracic echocardiography, and gastrointestinal endoscopy. Only Japanese patients hospitalized for initial evaluation of SSc were recruited into the present study. We excluded patients who did not meet the classification criteria for SSc established by American College of Rheumatology and European League Against Rheumatism in 2013 (ACR/EULAR criteria 2013) [24]. This study was approved by the ethics committee at The University of Tokyo Hospital.

### Data collection

We retrospectively reviewed the electronic medical records. The patients’ demographic information, laboratory results, and examination findings were obtained at the time closest to the first admission to our clinic. The collected demographic information included age, sex, disease duration, follow-up duration, and medication regimen. The laboratory results included autoantibody profiles, blood cell counts, C-reactive protein (CRP) levels, and erythrocyte sedimentation rates (ESRs).

Longitudinal data of mRSS and the result of pulmonary function test were collected from the most recent time when these examinations were performed at the same time.

### Qualitative evaluation of organ involvements

Systolic dysfunction of the heart was defined as left ventricle ejection fraction (LVEF) < 50%. Diastolic dysfunction of the heart was defined as ratio between early mitral inflow velocity and mitral annular early diastolic velocity (*E*/*e*’) > 15. Pulmonary hypertension was defined as mean pulmonary artery pressure > 25 mmHg on right heart catheterization. Presence of SSc-ILD was determined upon HRCT readings. Restrictive impairment of the lung was defined as the percentage of predicted forced vital capacity (%FVC) < 80%. Patients with the ratio of forced expiratory volume in 1 s to forced vital capacity (FEV1%) < 70% were excluded to rule out the obstructive components of the lung. Diffusion impairment of the lung was defined as the percentage of diffusing capacity for carbon monoxide (%DLco) < 70%. Classification of GERD findings from upper gastrointestinal endoscopy was performed by gastroenterologists, and reflux esophagitis was defined as Grade M or more on Los Angeles classification of esophagus [25]. Myositis was defined as elevation of serum CK twofold higher than the upper normal limit.

The reviewed disease-related events contained death, SRC, ileus, and heart failure that were observed since the primary evaluation until the most recent arrival to our clinic. Occurrence of SRC, ileus, and heart failure was determined upon clinical diagnosis.

### Quantitative or categorical evaluation of organ involvements

The degree of skin sclerosis was scored on mRSS by 6 dermatologists at The University of Tokyo Scleroderma Center, all of whom had been trained by repeated teaching method previously described [26]. The evaluation value of lung involvement included serum levels of Krebs von den Lungen-6 (KL-6) and surfactant protein-D (SP-D) levels, and pulmonary function parameters including %FVC and %DLco. Quantitative evaluation of heart involvement encompassed serum level of brain natriuretic peptide (BNP) and the results of echocardiography including LVEF, *E*/*e*’, and right ventricle systolic pressure (RVSP). Renal involvement was described by estimated glomerular filtration rate (eGFR). Severity of GERD on upper gastrointestinal endoscopy was categorically evaluated by Los Angeles classification of esophagus. Quantitative measurement of musculoskeletal system included the serum level of creatinine kinase (CK).

### Statistical analyses

Univariate analysis was performed by single logistic analysis for categorical variables and single regression analysis for continuous variables. Correlation analysis was also performed between some continuous variables to estimate Pearson’s correlation coefficient. Multivariate analysis was performed by multiple logistic analysis for categorical variables and multiple regression analysis for continuous variables. Explanatory variables in multivariate analysis were age, sex, disease duration, and mRSS. In sensitivity analyses, the following explanatory variables were added: baseline presence of pulmonary hypertension, the use of corticosteroids or immunosuppressants, the use of vasoactive agents (endothelin receptor antagonists, phosphodiesterase 5 inhibitors, beraprost, sarpogrelate hydrochloride, limaprost alfadex, and angiotensin-converting enzyme inhibitors), and the history of smoking. All the analyses were performed using Stata/IC 15 (StataCorp LLC, TX, USA).

## Results

### Demographics of the subject patients

**Fig. 1:**
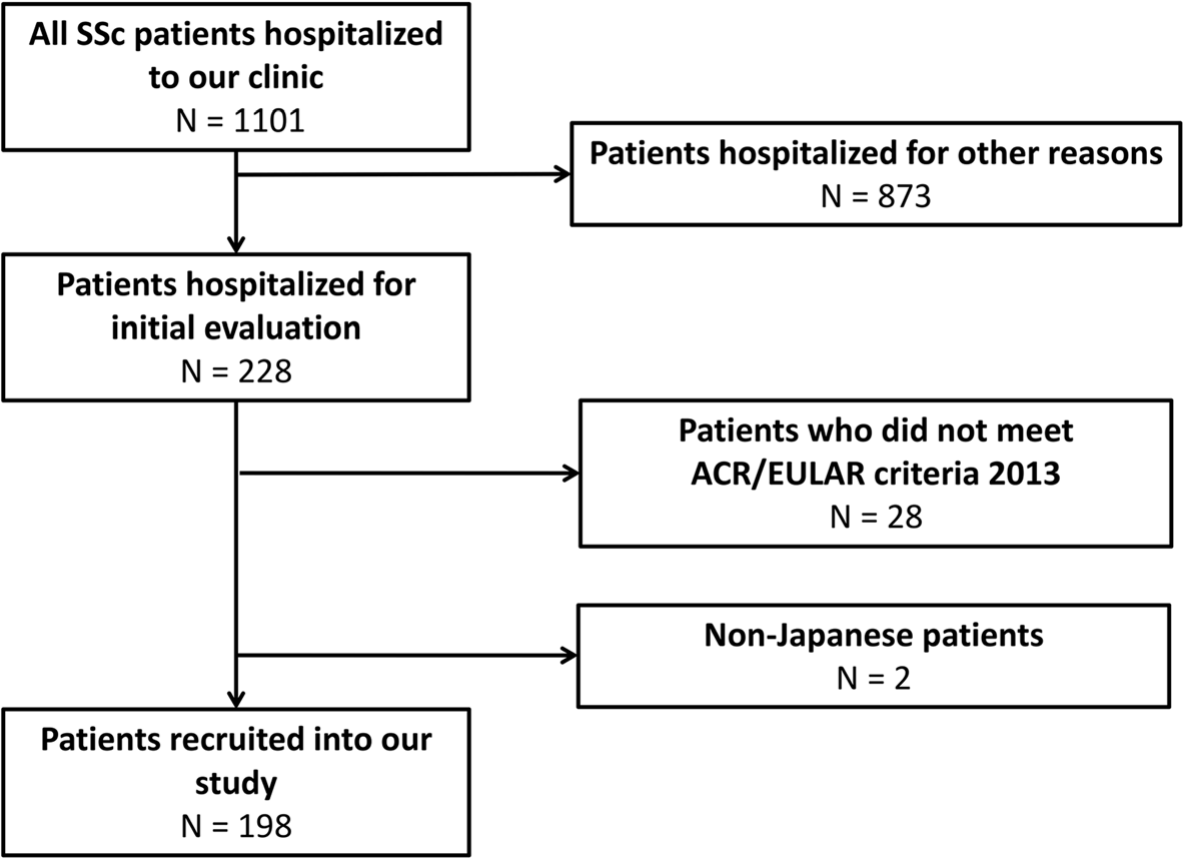
The flow chart of patient recruitment. All Japanese patients with systemic sclerosis (SSc) hospitalized to our clinic for initial evaluation were collected. Patients who did not meet the classification criteria for SSc established by American College of Rheumatology and European League Against Rheumatism in 2013 (ACR/EULAR criteria 2013) were excluded. In total, 198 patients were recruited into our study

In total, there were 1101 patients with SSc hospitalized to our clinic during the study period. We selected 228 patients who were hospitalized for initial evaluation of SSc and excluded 28 patients because they did not meet ACR/EULAR criteria 2013. Two patients were excluded because they were from Taiwan and the Philippines. As a result, 198 Japanese patients were recruited into our study (Fig. 1). The background features of the participants are summarized in Table 1. Their mean age was 55.4 years old (standard deviation [SD] = 15.5) with the number of female patients of 177 (89.4%). The mean disease duration was 7.3 years (SD = 8.8) with the mean follow-up duration of 3.2 years (SD = 2.3). The proportion of patients with history of smoking was 21.9%. Almost half of the patients (46.8%) had diffuse cutaneous SSc. The frequency of specific autoantibody positivity in the sera of the patients was as follows: anti-topoisomerase (topo) I antibody (Ab) in 78 patients (39.4%), anti-centromere Ab in 64 patients (32.3%), anti-RNA polymerase III Ab in 21 patients (10.6%), and anti-U1RNP Ab in 22 patients (11.1%). Corticosteroids or immunosuppressants had already been introduced in 50 patients (25.3%), and vasoactive agents had been administered to 74 patients (37.4%).

**Table 1:** Clinical demographics of the subject patients

	*N*	*n* (%) or mean (SD)
Male	198	21 (10.6%)
Female	198	177 (89.4%)
Age (years)	198	55.4 (15.5)
Disease duration (years)	196	7.3 (8.8)
Follow-up duration (years)	198	3.2 (2.3)
History of smoking	196	43 (21.9%)
Death	198	4 (2.0%)
Raynaud’s phenomenon	196	170 (86.7%)
Puffy finger	144	97 (67.4%)
Nail fold bleeding	190	139 (73.2%)
Telangiectasia	162	65 (40.1%)
Autoantibody		
Anti-topo I Ab	198	78 (39.4%)
Anti-centromere Ab	198	64 (32.3%)
Anti-RNA polymerase III Ab	198	21 (10.6%)
Anti-U1RNP Ab	198	22 (11.1%)
Medications		
Corticosteroids or immunosuppressants	198	50 (25.3%)
Corticosteroids	198	43 (21.7%)
Immunosuppressants	198	19 (9.6%)
Vasoactive agents	198	74 (37.4%)
Endothelin receptor antagonists	198	14 (7.1%)
Phosphodiesterase 5 inhibitors	198	5 (2.5%)
Beraprost	198	52 (26.3%)
Sarpogrelate hydrochloride	198	26 (13.1%)
Limaprost alfadex	198	15 (7.6%)
Angiotensin-converting enzyme inhibitors	198	3 (1.5%)
Others		
Non-steroidal anti-inflammatory drugs	198	27 (13.6%)
Tocopherol nicotinate	198	64 (32.3%)
Proton pump inhibitors	198	87 (43.9%)
Laboratory tests		
White blood cells (/mm^3^)	198	6700 (2300)
Hemoglobin (g/dL)	198	12.3 (1.9)
Hematocrit (%)	198	38.5 (5.1)
Platelets (× 10^4^/mm^3^)	198	26.7 (7.8)
CRP (mg/dL)	198	0.43 (1.19)
ESR (mm/h)	195	25.8 (19.7)
Skin involvement		
Qualitative measurement		
Diffuse cutaneous systemic sclerosis	190	89 (46.8%)
Quantitative measurement		
mRSS	177	9.9 (8.9)
Lung involvement		
Qualitative evaluation		
SSc-ILD	196	87 (44.4%)
Restrictive impairment	197	36 (18.3%)
Diffusion impairment	191	33 (17.3%)
Quantitative evaluation		
KL-6 (U/mL)	198	519 (499)
SP-D (ng/mL)	190	107 (98)
%FVC (%)	197	95.9 (20.8)
%DLco (%)	191	88.3 (20.0)
FEV1% (%)	197	82.1 (8.7)
Heart involvement		
Qualitative evaluation		
Systolic dysfunction	184	0 (0%)
Diastolic dysfunction	150	10 (6.7%)
Pulmonary hypertension	198	5 (2.5%)
Heart failure	198	3 (1.5%)
Quantitative evaluation		
BNP (pg/mL)	191	43.0 (50.0)
LVEF (%)	184	70.1 (6.3)
*E*/*e*’	150	9.6 (3.5)
RVSP (mmHg)	181	27.3 (7.5)
Renal involvement		
Qualitative evaluation		
SRC	198	6 (3.0%)
Quantitative evaluation		
eGFR (mL/min/1.73 m^2^)	198	87.8 (25.9)
Gastrointestinal involvement		
Qualitative evaluation		
Reflux esophagitis	179	78 (43.6%)
Ileus	198	6 (3.0%)
Categorical evaluation		
Los Angeles classification		
Grade N	179	101 (56.4%)
Grade M	179	30 (16.8%)
Grade A	179	32 (17.9%)
Grade B	179	11 (6.2%)
Grade C	179	4 (2.2%)
Grade D	179	1 (0.6%)
Musculoskeletal involvement		
Qualitative evaluation		
Myositis	197	7 (3.6%)
Quantitative evaluation		
CK (U/L)	197	110 (119)

*N* number of the observation, *n* number of the patients applicable, *SD* standard deviation

### Incidence and prevalence of organ involvements

The number of patients with each organ involvement was as follows: interstitial lung diseases in 87 patients (44.4%), restrictive impairment of the lung in 36 patients (18.3%), diffusion impairment of the lung in 33 patients (17.3%), diastolic dysfunction of the heart in 10 patients (6.7%), pulmonary hypertension in 5 patients (2.5%), heart failure in 3 patients (1.5%), SRC in 6 patients (3.0%), reflux esophagitis in 78 patients (43.6%), ileus in 6 patients (3.0%), and myositis in 7 patients (3.6%). There were no patients with systolic dysfunction of the heart.

### Single and multiple logistic analyses revealed that mRSS is associated with death, SRC, and lung involvement

Single logistic analyses revealed that higher mRSS is related to higher incidence of death (*P* < 0.05) and SRC (*P* < 0.05). Higher mRSS was also related to baseline presence of SSc-ILD (*P* < 0.05), restrictive impairment (*P* < 0.01), and diffusion impairment (*P* < 0.01) of the lung (Table 2). Multiple logistic analyses showed that relation between mRSS and these organ involvements is statistically significant even after compensating with the patients’ basic characteristics (sex, age, and disease duration). Meanwhile, no relationship was found between mRSS and incidence of ileus and heart failure. Furthermore, there was no relationship between mRSS and presence of reflux esophagitis and myositis. Collectively, higher mRSS was significantly and independently associated with higher incidence of death and SRC and higher prevalence of SSc-ILD, restrictive impairment, and diffusion impairment of the lung.

**Table 2:** Logistic analysis of relationship between mRSS and qualitative evaluation of organ involvement

	Univariate analysis		Multivariate analysis	
	*N*	OR (95% CI)	*N*	OR (95% CI)
Death	177	1.15* (1.03 to 1.29)	176	1.23* (1.03 to 1.46)
Lung involvement				
Interstitial lung disease	175	1.04* (1.00 to 1.08)	174	1.05* (1.01 to 1.09)
Restrictive impairment	176	1.07** (1.03 to 1.12)	175	1.07** (1.02 to 1.12)
Diffusion impairment	171	1.07** (1.02 to 1.11)	170	1.06* (1.01 to 1.11)
Heart involvement				
Diastolic dysfunction	136	1.03 (0.96 to 1.10)		
Pulmonary hypertension	177	0.97 (0.86 to 1.09)		
Heart failure	177	1.11 (0.99 to 1.23)		
Renal involvement				
SRC	177	1.11* (1.02 to 1.20)	176	1.11* (1.01 to 1.23)
Gastrointestinal involvement				
Reflux esophagitis	161	1.03 (0.99 to 1.07)		
Ileus	177	1.04 (0.95 to 1.13)		
Musculoskeletal involvement				
Myositis	177	1.03 (0.95 to 1.11)		

*N* number of the observation, *OR* odds ratio, *CI* confidence interval. Asterisk (*) indicates statistical significance in logistic analysis.*P < 0.05; **P < 0.01

### Single and multiple regression analyses showed the correlation between mRSS and lung involvement

**Fig. 2:**
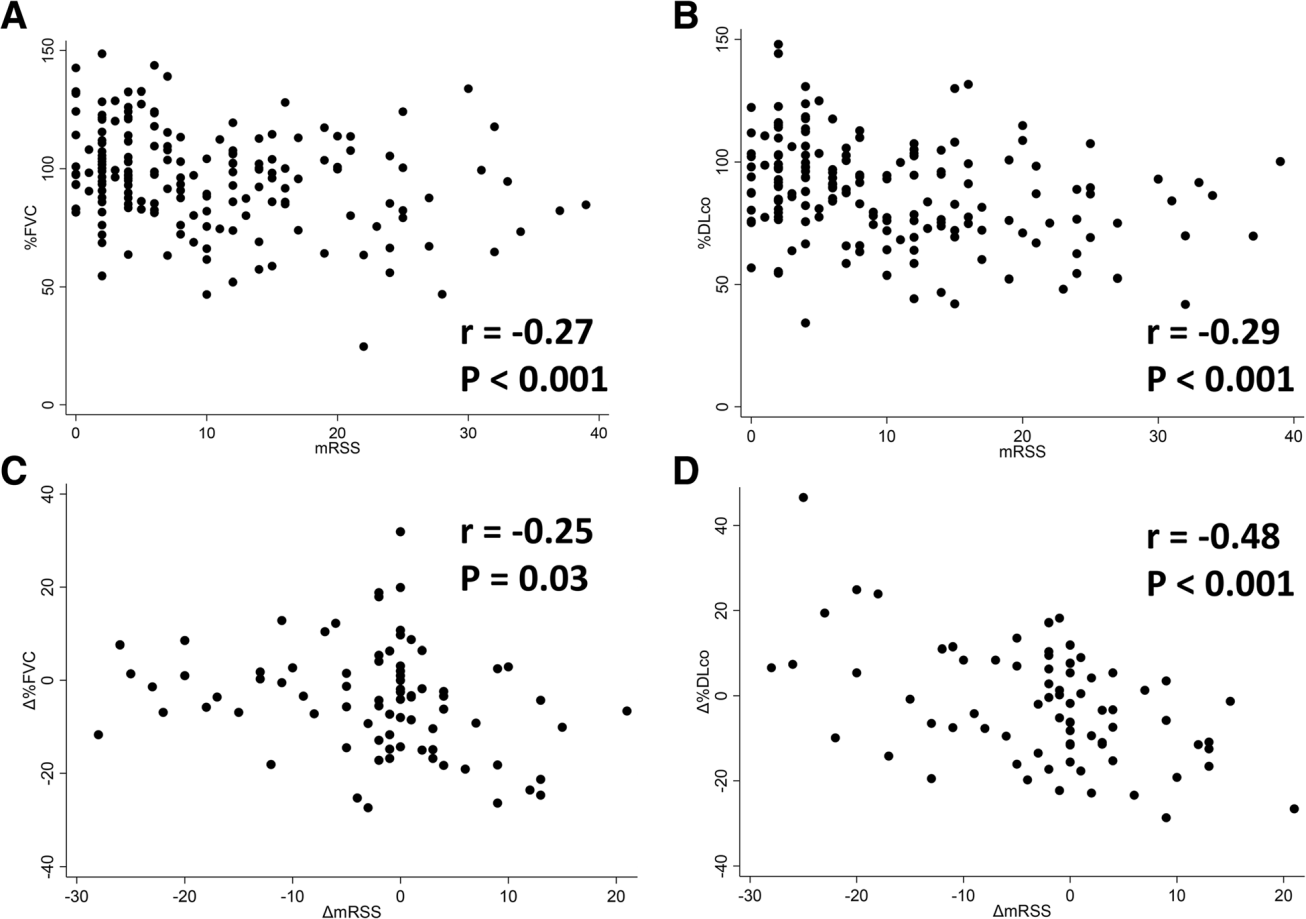
The scatter plot of mRSS and the result of pulmonary function test. On the baseline, **a** %FVC and **b** %DLco negatively correlate with mRSS (correlation coefficient [*r*] = − 0.27, *P* < 0.001 and *r* = − 0.29, *P* < 0.001, respectively). In addition, the longitudinal analysis showed negative correlation of ΔmRSS with **c** Δ%FVC and **d** Δ%DLco (*r* = − 0.25, *P* = 0.03 and *r* = − 0.48, *P* < 0.001, respectively)

Single regression analyses revealed that mRSS negatively correlates with %FVC (*P* < 0.001) and %DLco (*P* < 0.001) and positively correlates with eGFR (*P* < 0.05) and the serum level of SP-D (*P* < 0.05; Table 3). Correlation analyses also showed negative correlation between mRSS and %FVC (*P* < 0.001; Fig. 2a) or %DLco (*P* < 0.001; Fig. 2b). There was no correlation between mRSS and the serum levels of KL-6 and CK, the results of echocardiography, or endoscopic findings in the esophagus.

**Table 3:** Regression analysis of correlation between mRSS and quantitative or categorical evaluation of organ involvement

	Univariate analysis		Multivariate analysis	
	*N*	*β* (95% CI)	*N*	*β* (95% CI)
Lung involvement				
KL-6	177	5.14 (−3.38 to 13.7)		
SP-D	170	1.87* (0.27 to 3.47)	169	1.87* (0.09 to 3.65)
%FVC	176	− 0.64*** (− 0.98 to − 0.30)	175	− 0.61** (− 0.98 to − 0.23)
%DLco	171	− 0.67*** (− 1.00 to − 0.33)	170	− 0.53** (− 0.89 to − 0.16)
Heart involvement				
BNP	171	0.82 (− 0.02 to 1.66)		
LVEF	166	0.06 (− 0.05 to 0.17)		
*E*/*e*’	136	0.02 (− 0.04 to 0.09)		
RVSP	164	0.11 (− 0.02 to 0.25)		
Renal involvement				
eGFR	177	0.44* (0.03 to 0.85)	176	0.11 (− 0.25 to 0.46)
Gastrointestinal involvement				
Los Angeles classification	161	0.02 (− 0.01 to 0.04)		
Musculoskeletal involvement				
CK	177	1.99 (− 0.09 to 4.07)		

*N* number of the observation, *β* regression coefficient, *CI* confidence interval. Asterisk (*) indicates statistical significance in regression analysis.*P < 0.05; **P < 0.01; ***P < 0.001

In addition, multiple regression analyses showed that negative correlation between mRSS and the results of pulmonary function test or serum levels of SP-D is statistically significant even after compensation with the patients’ basic characteristics. In contrast, correlation between mRSS and eGFR was not statistically significant in multiple regression analyses.

### Sensitivity analyses clarified that correlation between mRSS and pulmonary function is independent from other explanatory variables

Sensitivity analysis of the multivariate regression model was performed by adding other explanatory variables. The significance of mRSS as an explanatory variable of %FVC and %DLco was robust to adding baseline presence of pulmonary hypertension, the use of corticosteroids or immunosuppressants, the use of vasoactive agents, or the history of smoking (Table 4). Thus, mRSS significantly and independently correlated with %FVC and %DLco.

**Table 4:** Sensitivity analysis of the multivariate regression model

Additional variables	%FVC vs mRSS		%DLco vs mRSS	
	*N*	*β* (95% CI)	*N*	*β* (95% CI)
Pulmonary hypertension	175	− 0.59** (− 0.97 to − 0.22)	170	− 0.52** (− 0.88 to − 0.16)
Use of corticosteroids or immunosuppressants	175	− 0.52** (− 0.89 to − 0.15)	170	− 0.45* (− 0.81 to − 0.10)
Use of vasoactive agents	175	− 0.59** (− 0.97 to − 0.22)	170	− 0.50** (− 0.85 to − 0.14)
History of smoking	174	− 0.62** (− 0.98 to − 0.27)	169	− 0.52** (− 0.88 to − 0.16)

*β* regression coefficient, *CI* confidence interval. Asterisk (*) indicates statistical significance in regression analysis.*P < 0.05; **P < 0.01

### Subgroup analyses revealed correlation between mRSS and lung function is significant in patients with anti-topo I Ab and in patients with disease duration shorter than 5 years

We performed subgroup analyses of correlation between mRSS and baseline %FVC or %DLco by autoantibody profile and disease duration (Table 5). In patients with anti-topo I Ab, the correlation to %FVC and %DLco was statistically significant in both single regression analyses (%FVC: *P* < 0.05; %DLco: *P* < 0.01) and multiple regression analyses (%FVC: *P* < 0.01; %DLco: *P* < 0.05). By contrast, these correlations were not statistically significant among patients with anti-centromere, anti-RNA polymerase III, or anti-U1 RNP Abs. In addition, multivariate regression analysis showed significant correlation between mRSS and both %FVC (*P* < 0.05) and %DLco (*P* < 0.05) in SSc patients with disease duration shorter than 5 years, while mRSS significantly correlated with %FVC (*P* < 0.01) but not with %DLco among patients with disease duration of 5 years or more. Taken together, correlation between mRSS and %FVC or %DLco was significant especially among patients with anti-topo I Ab and patients with disease duration shorter than 5 years.

**Table 5:** Subgroup analysis of correlation between mRSS and pulmonary function by autoantibody profile or disease duration

	Univariate analysis		Multivariate analysis	
	*N*	*β* (95% CI)	*N*	*β* (95% CI)
%FVC				
Autoantibody profile				
Anti-topo I Ab	65	− 0.68* (− 1.25 to − 0.11)	65	− 0.80** (− 1.39 to − 0.20)
Anti-centromere Ab	60	− 1.36 (− 1.18 to 0.91)		
Anti-RNA polymerase III Ab	17	0.66 (− 0.29 to 1.61)		
Anti-U1RNP Ab	19	− 0.09 (− 1.07 to 0.89)		
Disease duration				
< 5 years	106	− 0.41* (− 0.77 to − 0.05)	106	− 0.43* (− 0.84 to − 0.02)
≥ 5 years	69	− 1.53*** (− 2.36 to − 0.70)	69	− 1.48** (− 2.33 to − 0.63)
%DLco				
Autoantibody profile				
Anti-topo I Ab	62	− 0.80** (− 1.36 to − 0.24)	53	− 0.68* (− 1.27 to − 0.08)
Anti-centromere Ab	58	− 0.02 (− 1.02 to 1.07)		
Anti-RNA polymerase III Ab	17	− 0.07 (− 1.03 to 0.89)		
Anti-U1RNP Ab	19	− 0.63 (− 1.59 to 0.32)		
Disease duration				
< 5 years	104	− 0.70** (− 1.10 to − 0.30)	104	− 0.52* (− 0.97 to − 0.07)
≥ 5 years	66	− 0.86* (− 1.61 to − 0.11)	66	− 0.68 (− 1.43 to 0.06)

*N* number of the observation, *β* regression coefficient, *CI* confidence interval. Asterisk (*) indicates statistical significance in regression analysis.*P < 0.05; **P < 0.01; ***P < 0.001

### Longitudinal analyses showed negative correlation between the change in mRSS and that in %FVC and %DLco

Longitudinal data was available for 84 patients (42.4%). The mean follow-up duration among those patients was 2.5 years (SD = 1.9). We examined the correlation between mRSS change (ΔmRSS) and pulmonary function change (Δ%FVC and Δ%DLco). Correlation analyses showed that ΔmRSS negatively correlated with both Δ%FVC (*P* = 0.03; Fig. 2c) and Δ%DLco (*P* < 0.001; Fig. 2d). Thus, the longitudinal change in mRSS negatively correlated with the longitudinal change in %FVC and %DLco.

## Discussion

Our retrospective observation of SSc patients revealed that mRSS significantly correlates with quantitative measurements of the lung involvement such as %FVC and %DLco on the baseline. The correlation in multivariate regression analysis was robust to adding baseline presence of pulmonary hypertension, the use of corticosteroids or immunosuppressants, the use of vasoactive agents, and the history of smoking as explanatory variables. Moreover, the longitudinal change in mRSS significantly correlated with that in %FVC and %DLco. Although previous studies have shown that higher skin thickness score is related to the existence of organ involvements [15–19], correlation between skin thickness score and quantitative barometers of each organ involvement has not yet been documented in Japan. This is the first study that revealed correlation between skin thickness score and quantitative measurements of organ involvements in Japanese SSc patients.

Close relationship between skin sclerosis and lung fibrosis in SSc patients is suggested by several aspects of clinical experience. First, skin sclerosis and SSc-ILD share their chronology; they both develop in the first few years in the natural time course of SSc [27]. This corresponds to our result that correlation between skin score and pulmonary function was prominent in patients with shorter disease duration. Second, pathohistological feature of skin involvement and lung involvement in SSc patients is quite similar; invasion of inflammatory cells is seen in their early stage, and proliferation and degeneration of collagen fibers is observed in their late stage [2, 3]. Third, SSc patients with anti-topo I Ab experience combination of severe skin sclerosis and SSc-ILD [7, 8]. Indeed, correlation between mRSS and pulmonary function was prominent in patients with anti-topo I Ab in our study. It suggests that skin and lung fibrosis in SSc has similar abnormality of immune system as its background. Forth, recent clinical experiences have indicated that both skin and lung fibrosis responds well to B cell-targeting therapy, including rituximab and tocilizumab. Previously, our group has revealed that B cells play a key role in the pathogenesis of SSc [28]. Abnormality of B cell function including production of autoantibodies and inflammatory cytokines, such as interleukin-6 (IL-6), contributes to the progression of fibrosis in SSc mouse models [29]. Rituximab, a chimeric monoclonal Ab binding to CD20, ablates B cells from blood circulation via targeting CD20 expressed on the surface of B cells. Some open-label clinical studies [30–33] and a retrospective case-control study [34] revealed that SSc patients on rituximab showed significant improvement of mRSS and %FVC, which is now being verified by an ongoing double-blind randomized placebo-controlled trial (UMIN000030139). Tocilizumab, a humanized monoclonal Ab binding to IL-6 receptors, inhibits the signaling pathway via IL-6 mainly secreted by B cells that modulate inflammation and tissue fibrosis [35, 36]. A double-blind randomized placebo-controlled trial (NCT01532869) indicated that weekly subcutaneous injection of tocilizumab reduces mean mRSS and prevents FVC from decline [37, 38]. These facts highlight the crucial role of B cells in the pathogenesis of both skin and lung involvement of SSc.

Furthermore, our study showed that higher mRSS is predictive for higher mortality and higher incidence of SRC. These results from Japanese SSc cohort are consistent with those of previous studies in other ethnic populations [18, 19], which indicates that skin thickness may be an indicator of not only organ fibrosis represented by skin fibrosis and SSc-ILD, but also other aspects of SSc such as vasculopathy.

From a viewpoint of clinical application, mRSS is promised as a good surrogate marker of lung involvement in SSc patients. Our study showed that mRSS significantly correlates with %FVC and %DLco in SSc patients both on the baseline and along the time course. Other evaluation methods for SSc-ILD, including serologic marker measurements, HRCT, or 6-min walk test, are more invasive or time-consuming than measuring mRSS. Moreover, it has also been reported that mRSS is a measurement tool with less inter- and intra-observer variation [39]. Skin thickness score might be a reasonable way of monitoring the efficacy of treatments for SSc-ILD in both clinical trials and real clinical settings. Furthermore, mRSS possibly reflects the global disease activity and severity of SSc. Indeed, Composite Response Index for Systemic Sclerosis includes mRSS in its scoring [40]. Taken together, although further studies are warranted, evaluation of mRSS is useful as a surrogate marker of organ involvements, global disease activity, and severity in SSc.

The major limitation of the present study is its retrospective design. There is some missing in the data especially in longitudinal data, which may bias the result of the analyses. Moreover, the small sample size and short observation term leads to the low power of the analysis, especially for rare disease-related events or subgroup analyses. Prospective observational studies with bigger sample size and longer follow-up are desirable. In addition, the present study includes only Japanese patients. The prevalence of SSc-ILD, decreased %DLco [41], pulmonary hypertension [42], and esophagitis [43] was lower than those reported in previous studies in other populations. Validation of our study should be conducted targeting other ethnicities in the future.

## Conclusions

Retrospective observation of Japanese SSc patients revealed significant correlation between skin thickness and pulmonary function. Skin thickness score is a promising surrogate marker of lung involvement in systemic sclerosis.

## Data Availability

The datasets used and/or analyzed during the current study are available from the corresponding author on reasonable request.

## Abbreviations

%DLco: Percentage of predicted diffusing capacity of the lungs for carbon monoxide
%FVC: Percentage of predicted forced vital capacity
95% CI: 95% confidence interval
Ab: Antibody
ACR: American College of Rheumatology
BNP: Brain natriuretic peptide
CK: Creatinine kinase
CRP: C-reactive protein
*E*/*e*’: Ratio between early mitral inflow velocity and mitral annular early diastolic velocity
eGFR: Estimated glomerular filtration rate
ESR: Erythrocyte sedimentation rate
EULAR: European League Against Rheumatism
FEV1%: The ratio of forced expiratory volume in 1 s to forced vital capacity
GERD: Gastroesophageal reflux disease
HRCT: High-resolution computed tomography
IL-6: Interleukin-6
ILD: Interstitial lung disease
KL-6: Krebs von den Lungen-6
LVEF: Left ventricle ejection fraction
mRSS: Modified Rodnan total skin thickness score
*N*: Number of the observation
*n*: Number of the patients applicable
OR: Odds ratio
*r*: Correlation coefficient
RVSP: Right ventricle systolic pressure
SD: Standard deviation
SP-D: Surfactant protein-D
SRC: Systemic sclerosis renal crisis
SSc: Systemic sclerosis
*β*: Regression coefficient
